# Impact of ILD-Specific Therapies on Perioperative Course in Patients with Progressive Interstitial Lung Disease Undergoing Lung Transplantation

**DOI:** 10.3390/jcm12154996

**Published:** 2023-07-29

**Authors:** Dieter Munker, Paola Arnold, Gabriela Leuschner, Michael Irlbeck, Sebastian Michel, Teresa Kauke, Bruno Meiser, Jürgen Behr, Nikolaus Kneidinger, Tobias Veit

**Affiliations:** 1 Department of Medicine V, University Hospital LMU Munich, Comprehensive Pneumology Center (CPC-M), Member of the German Center for Lung Research (DZL), 81377 Munich, Germany; dieter.munker@med.uni-muenchen.de (D.M.); paola.arnold@med.uni-muenchen.de (P.A.); juergen.behr@med.uni-muenchen.de (J.B.); nikolaus.kneidinger@med.uni-muenchen.de (N.K.); 2 Department of Anaesthesiology, University of Munich (LMU), 81377 Munich, Germany; michael.irlbeck@med.uni-muenchen.de; 3 Clinic of Cardiac Surgery, University of Munich (LMU), 81377 Munich, Germany; sebastian.michel@med.uni-muenchen.de; 4 Department of Thoracic Surgery, University of Munich (LMU), 81377 Munich, Germany; teresa.kauke@med.uni-muenchen.de; 5 Transplant Center, University of Munich, 81377 Munich, Germany

**Keywords:** lung transplantation, idiopathic pulmonary fibrosis, progressive pulmonary fibrosis, antifibrotic treatment, immunosuppression, posttransplant outcome

## Abstract

Immunosuppressants and antifibrotics are currently used to treat patients with various interstitial lung diseases, which may undergo lung transplantation (LTx). The retrospective study aimed to evaluate the potential effects of therapeutic regimen on the perioperative course in patients with idiopathic pulmonary fibrosis (IPF) or progressive pulmonary fibrosis (PPF) undergoing LTx. All patients with IPF and PPF undergoing LTx between January 2014 and December 2021 were included. We retrospectively screened for previous use of immunosuppressants and antifibrotic therapy. We analyzed perioperative courses, short-term outcomes, and safety retrospectively. In total, 286 patients with diagnosis of IPF or PPF were analyzed. According to the treatment regimen before LTx, the study cohort was divided into four groups and compared. No differences between antifibrotic monotherapy, combined antifibrotic and immunosuppressive therapy with regard to postoperative complications were observed. Length of mechanical ventilation was shorter in patients with antifibrotics prior to LTx. Pretreatment with antifibrotic monotherapy and a combination of antifibrotic drugs with immunosuppressive therapy, lower body mass index (BMI) and lower blood loss, were independently associated with primary graft dysfunction grades 0–3 72 hours after LTx (*p* < 0.001). Finally, patients with antifibrotic monotherapy developed significantly less de novo donor-specific antibodies (DSA) (*p* = 0.009). Higher intraoperative blood loss, etiology of interstitial lung disease (ILD) and older age were independently associated with shorter survival after LTx. Use of antifibrotic monotherapy and a combination of antifibrotic drugs with immunosuppressive therapy in IPF/PPF patients undergoing LTx, proved to be safe and might lead to beneficial effects after LTx.

## 1. Introduction

Progressive pulmonary fibrosis (PPF) and idiopathic pulmonary fibrosis (IPF) are associated with irreversible loss of lung function and major reductions in quality of life and survival regardless of the underlying condition [1,2,3]. Therefore, for selected patients, lung transplantation can be offered as the last treatment option after failure of medical therapy in severe ILD.

Survival after lung transplantation is limited by non-allograft-related and allograft-related factors. The major challenge in the postoperative phase is to gain control of immunological reactions and to reduce ischemia-reperfusion injury. Those factors can contribute to acute systemic inflammation, alter the transplanted lungs and lead to primary graft dysfunction (PGD) and thus to prolonged ventilation and adverse outcomes [4].

Recently, the treatment landscape for fibrotic lung diseases has changed after randomized trials demonstrated clinical efficacy of antifibrotic therapy in patients with PPF. Furthermore, two antifibrotic medications, pirfenidone, and nintedanib, first approved for idiopathic pulmonary fibrosis (IPF), have shown consistent effects on lung function decline in several diseases of non-IPF-patients [5,6,7,8]. Therefore, a new treatment pathway for PPF is provided, and combined antifibrotic and immunosuppressant drug therapy has become a relevant treatment option [3].

Still, experience with patients undergoing lung transplantation (LTx) with prior treatment of immunosuppressants, antifibrotics or a combination of both is scarce. Most data are limited to patients pretreated with prednisolone monotherapy or IPF patients pretreated with antifibrotics [9,10,11,12,13,14,15].

This study aimed to evaluate the potential effects of different treatment regimens on post-transplant outcomes and immunology in patients with IPF and PPF after LTx in a retrospective cohort.

## 2. Methods

### 2.1. Study Cohort and Design

All transplant candidates with IPF and PPF who underwent LTx from January 2014 until December 2021 were included in the study and analyzed retrospectively. The time point was chosen since, in 2014, nintedanib, the second antifibrotic drug, was approved for IPF. The study was performed at the University of Munich, Germany, and approved by the local ethics committee (UE No. 646-16).

Individuals with a consensus diagnosis of ILD such as IPF, fibrotic hypersensitivity pneumonitis (HP), non-specific interstitial pneumonia (NSIP), autoimmune ILDs such as rheumatoid arthritis-associated ILD, SSc-ILD, mixed connective tissues disease-associated ILD, rare ILDs and unclassified pulmonary fibrosis (uILD) were included. All diagnoses were made in accordance with the current international criteria [16,17,18,19,20]. Diagnosis was verified by histological examination of explanted lungs.

Local databases and medical records were screened for therapy with antifibrotic medication and/or immunosuppression prior to transplantation. The study population was divided into 4 groups according to the treatment regimen prior to LTx: antifibrotic monotherapy (nintedanib or pirfenidone); combination therapy of antifibrotic drugs with immunosuppression; immunosuppressive treatment; and without specific medication. Antifibrotics were applied until at least one week prior to transplantation. Treatment with nintedanib or pirfenidone was not continued after LTx. Immunosuppressive drugs included corticosteroids, azathioprine, mycophenolate mofetil or methotrexate. If indicated, immunosuppressive medication was continued until transplantation. After LTx, all patients received standard immunosuppressive triple therapy including corticosteroids, mycophenolate mofetil and calcineurin inhibitors. [13].

All patients were screened to identify important comorbidities and contraindications before transplantation. To assess the severity of disease, we performed the lung allocation score (LAS) score, gender-age-physiology-ILD-index (GAP-ILD), lung function analysis including blood gas analysis, spirometry, plethysmography and 6-min walking distance (6MWD) according to the German LAS business rules [21]. Baseline characteristics were collected at the time of entering the waiting list.

### 2.2. Short- and Long-Term Outcomes

We used Narkodata (online-documentation system, Imeso GmbH, Giessen, Germany) to collect perioperative data, including the procedure duration, blood loss and use of extracorporeal membrane oxygenation therapy (ECMO). Further postoperative data included length of intensive care unit (ICU) stay, hours of mechanical ventilation, primary graft dysfunction (PGD), need of reintubation and revision surgeries. We defined revision surgeries as surgery during hospital stay due to postoperative bleeding, wound infection or healing disorder.

PGD was assessed at 72 h after LTx (defined as T72) according to the ISHLT guidelines. PGD was classified into three grades (grade 1 > 300 PaO_2_/FiO_2_; grade 2 200 to 300 PaO_2_/FiO_2_; grade 3 < 200 PaO_2_/FiO_2_) based on the ratio of partial pressure of oxygen (PaO_2_)/fraction of inspired oxygen (FiO_2_). Absence of pulmonary edema on chest radiograph of the transplanted lung was defined as grade 0. Any patient using a nasal cannula for oxygen or Venturi mask was graded as 0 or 1, as described previously [13]. Any patient receiving extracorporeal membrane oxygenation or mechanically ventilated with an FiO_2_ of greater than 0.5 on nitric oxide beyond 72 h after transplantation was considered grade 3 [22].

Transbronchial lung biopsies (TBB) and screening for human leukocyte antigen (HLA)-antibodies against HLA class I and II (Luminex, Life Screen Deluxe, Gen-Probe, USA) were routinely performed within 30 days after LTx and if clinically indicated. TBB were classified as A0 to A4 according to International Society of Heart and Lung Transplantation (ISHLT) guidelines [23,24]. Bronchoalveolar lavage (BAL) was performed after 3, 6 and 12 months if clinically indicated [25]. Additionally, mortality after LTx was recorded during the follow up of at least one year after LTx. Causes of death in the study cohort were assessed based on medical records.

### 2.3. Statistical Analysis

Continuous variables are presented as the mean ± standard deviation (SD), with categorical variables summarized by frequency and percentage. Analysis of variance (ANOVA) was used to compare continuous variables; significant differences identified on preliminary testing between the groups were analyzed separately by post hoc pairwise comparisons using Tukey’s honest significant difference test. Fisher’s exact test was used to compare categorical variables.

To identify possible associations of certain variables on outcome parameters we first used univariate analysis to compare the distribution of variables between groups. Second, we performed logistic regression analysis to model multivariate associations of possible predictors. Predictors for the regression models were selected with regard to significance in the univariate analysis and under consideration of the rules regarding overfitting and multicollinearity. In addition, multivariate cox regression analysis was used to evaluate the impact of different variables on survival over the entire follow-up. *p* < 0.05 was considered statistically significant. Data were statistically analyzed by SPSS version 24.0 (IBM SPSS, Armonk, NY, USA) statistical software.

## 3. Results

### 3.1. Study Cohort and Baseline Characteristics

**Figure 1:**
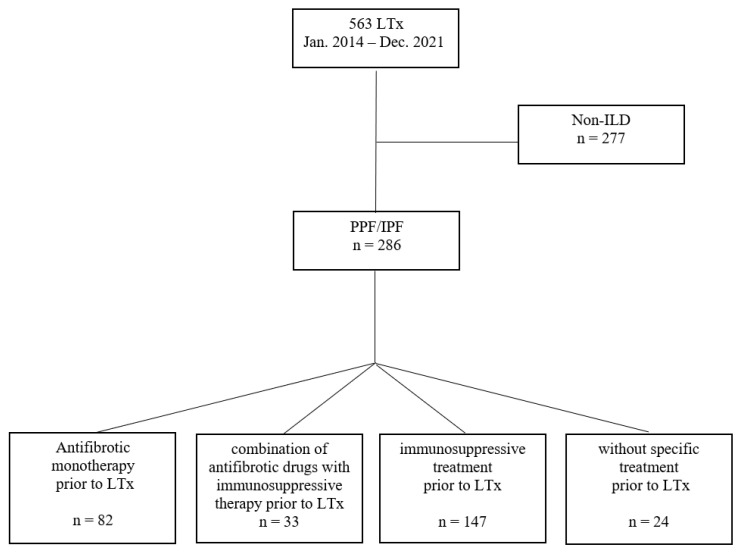
Description of the study cohort and classification. IPF: idiopathic pulmonary fibrosis; LTx: lung transplantation; PPF: progressive pulmonary fibrosis.

A total of 563 lung transplantations were performed during the study period. Thereof, 286 patients (50.8%) had an underlying diagnosis of end-stage ILD, including 170 lung transplant recipients with PPF (59.4%) and 116 patients with IPF (40.6%). The study cohort was divided into four groups as depicted in Figure 1—“antifibrotic monotherapy” (*n* = 82), “combination of antifibrotic drugs with immunosuppressive therapy” (*n* = 33), “immunosuppressive treatment” (*n* = 147) and “without specific treatment” (*n* = 24)—and analyzed. In the two groups with antifibrotic pretreatment, 108 patients (95.9%) took nintedanib or pirfenidone on the day of transplantation, whereas requirement of mechanical ventilation and/or ECMO therapy after ICU admission led to discontinuation of antifibrotic therapy in 7 patients (6.1%) two to seven days before surgery. In the immunosuppressive treatment group, 17 LTx recipients (5.9%) had previously received antifibrotic agents (13 pirfenidone only, 3 nintedanib only, 1 both). In these cases, antifibrotic medication was stopped due to progression of disease or side effects with a minimum of 2.5 months before transplantation. A detailed overview of the concomitant immunosuppressive therapy prior to LTx is shown in the supplementary material (Table S1). The most common therapy among the study cohort (28.0%) was pretreatment with corticosteroid monotherapy at a median dose of 5 mg/day (2.5–70 mg/day).

Characteristics of the study cohort, severity of disease and last LAS are demonstrated in Table 1. At the time of LTx, the groups with antifibrotic pretreatment were significantly older and had larger amounts of male patients and past smokers, as well as a higher proportion of IPF as the underlying disease than the immunosuppressive group. Severity of disease did not differ between the study groups. However, comorbidities assessed during transplant evaluation showed a higher prevalence of coronary artery disease (CAD) with a history of revascularization (22.0% vs. 3.0% vs. 8.2% vs. 8.3%, *p* = 0.007) in the group with antifibrotic monotherapy before LTx (Table S2).

### 3.2. Perioperative Course, ICU Parameters and Surgery-Related Complications

Acute respiratory worsening led to ICU admission with the need for mechanical ventilation in 15 patients (5.2%) and veno-venous ECMO therapy in 22 patients (7.7%) before LTx, respectively. No significant differences were observed between the groups (Table 2). Intraoperatively, the number of patients supported by venovenous or venoarterial ECMO was similar (4.9% vs. 3.1% vs. 10.3% vs. 12.5%, *p* = 0.301 and 51.2% vs. 56.3% vs. 58.2% vs. 50.0%, *p* = 0.718; respectively) as shown in Table 2. Intraoperative blood loss was increased in the study groups with immunosuppressive treatment or without any specific treatment prior to LTx (2105.7 ± 1409.7 mL vs. 2481.8 ± 1432.5 mL vs. 3238.6 ± 2532.2 vs. 3687.5 ± 2758.9 mL, *p* < 0.001). Operation time was not statistically different between the groups. Most of the patients (70.6%) could be successfully weaned from intraoperative ECMO therapy. In total, 37 patients (13.0%) received vv-ECMO, and 16 patients received va-ECMO (5.6%), respectively.

Furthermore, revision surgery, pneumothoraces and anastomotic complications were similar in patients with different pretreatments, as depicted in Table 2. Length of mechanical ventilation was shorter (59.1 h vs. 57.4 h vs. 98.0 h vs. 69.3 h, *p* = 0.019), and severity of PGD at 72 h after transplantation was lower in the groups pretreated with antifibrotic drugs (0.9 ± 0.9 vs. 0.8 ± 0.1 vs. 1.5 ± 1.1 vs. 1.7 ± 1.0, *p* < 0.001). Seventy-two hours after transplantation, PGD grade 3 was more frequent in the groups with immunosuppressive or no specific treatment before LTx (7.5% vs. 3% vs. 26.4% vs. 29.2%, *p* < 0.001) (Table 2). There was no difference regarding the rate of reintubation and the use of inhaled nitric oxide between the groups. Additionally, length of ICU stay was shorter (18.1 ± 42.0 vs. 14.0 ± 14.7 vs. 25.6 ± 34.0 vs. 25.6 ± 26.4, *p* = 0.196) in the group with the combination of antifibrotic drugs and immunosuppressive therapy.

Univariate and multivariate regression analysis was performed to take a deeper view into the diversity of treatment options prior to LTx and to identify the associations of certain variables on post-transplant outcome parameters (Table 3). Pretreatment with antifibrotic therapy, combination of antifibrotic drugs with immunosuppression, lower BMI and less intraoperative blood loss independently correlated with improved primary graft function after 72 h after LTx in multivariate regression analysis (*p* < 0.001) (Table 3).

### 3.3. Pre-Transplant Treatment and Outcome

Two patients (1.8%) in the immunosuppressive treatment group and one patient each in the groups with antifibrotic pretreatment (1.9%) received induction therapy with ATG. The number of patients with one or more episodes of acute cellular rejection (≥A1) within 30 days after transplantation was lower after treatment with antifibrotics (7.7% vs. 15.6% vs. 13.8% vs. 31.6%, p = 0.057) (Table 4). The prevalence of de novo donor-specific HLA-antibodies (DSA) within 30 days after transplantation was significantly higher in the groups without antifibrotic pretreatment (12.5% vs. 15.2% vs. 29.6% vs. 34.8%, *p* = 0.009). Pretreatment with antifibrotic monotherapy (*p* = 0.034; Coeff. ß: −0.227) was independently associated with lower risk for development of DSA within the first year after LTx while controlling for age, sex, BMI, pre- or postoperative ECMO therapy, etiology of ILD (IPF vs. Non-IPF), CTD-ILD, pulmonary arterial pressure, time from diagnosis to LTx, prednisolone dose > 5 mg, absence of specific treatment prior to LTx, combination of antifibrotic drugs and immunosuppressive therapy, intraoperative blood loss and LAS in regression analysis (*p* = 0.047).

Analysis of BAL assessed within the first year after LTx regarding neutrophilia was similar between the groups (Table 4).

During the first year after LTx 24 patients (8.4%) died (antifibrotic monotherapy 7.3%, combination of antifibrotic drugs with immunosuppressive therapy 3.0%, immunosuppressive treatment 10.9% and without specific treatment 4.2%; *p* = 0.516). The most common causes of death were septic shock and multiple-organ failure (*n* = 11), myocardial infarction or cardiogenic shock (*n* = 5) and COVID-19 pneumonia (*n* = 2). Other causes of death were primary transplant failure (*n* = 2), adenocarcinoma (*n* = 1), ischemic stroke (*n* = 1), pneumonia in CLAD (*n* = 1) and invasive aspergillosis (*n* = 1). None of the patients with combined antifibrotic and immunosuppressant drug therapy and in the group without specific treatment prior to LTx died during ICU stay. The overall survival rates for the study cohort were 97.9% at one month, 97.2% at six months and 91.6% at 12 months, respectively. Long-term survival over the entire study period, calculated using Kaplan–Meier estimates, showed no significant difference between the 4 treatment groups (*p* = 0.690) (Figure S1).

Intraoperative blood loss (Odd ratio 1.000, CI 1.000-1.000; *p* = 0.006), etiology of ILD (IPF vs. PFF) (Odd ratio 3.152, CI 1.463–6.790; *p* = 0.003), age (Odd ratio 1.072, CI 1.022–1.124; *p* = 0.004) and LAS score (Odd ratio 1.032, CI 1.010–1.054; *p* = 0.004) were associated with shorter survival. Still, no significant differences between pretransplant treatment regimens were revealed according to cox regression analysis (Table 5).

## 4. Discussion

Recently, the treatment landscape for the entire field of fibrotic lung diseases has changed after randomized trials demonstrated clinical efficacy of antifibrotic therapy in patients with IPF and PPF [3,5,6,7,8]. As a result, the therapeutic options for ILD patients prior to lung transplantation are constantly increasing. Our cohort study includes a large cohort of ILD patients with various immunomodulatory and antifibrotic therapies.

Except for prednisolone, there are no data on the impact of immunomodulating agents on LTx outcomes in patients with PPF. Prior studies showed that high dose of prednisolone therapy before transplantation might contribute to marked wound healing disorder, anastomotic dehiscence and mortality [14,15]. While the tolerable dose of prednisolone appears to be below 0.30–0.42 mg·kg^−1^·day^−1^, most centers consider a maintenance dose of <20 mg as eligible for LTX [14]. Interestingly, according to our data pretreatment with prednisolone (daily dose <10 mg) has no significant impact on PGD after 72 h and survival after LTX in regression analysis.

The effects of treatment with other immunosuppressive agents prior to LTX on lung transplantation outcomes are not well studied. In contrast to earlier studies, we included azathioprine, methotrexate and mycophenolate mofetil used to treat PPF. According to our data, anastomotic complications and operative revision weren’t significantly increased in patients pretreated with immunosuppressive medication. Compared to the other subgroups, patients with immunosuppressive therapy prior to LTx developed significantly more HLA-antibodies one month after LTX and longer duration of mechanical ventilation. These findings might be explained by the pathomechanisms of diseases with autoimmune features.

Previous therapy with the antifibrotic drugs may potentially interfere with immunological reactions and perioperative complications in ILD patients undergoing LTx.

In our cohort study, 40.2% of the lung transplant candidates received antifibrotic medication. In addition, 95.9% of the study cohort with previous antifibrotic treatment have taken nintedanib or pirfenidone until at least one week before transplantation, indicating a good adherence of antifibrotics in patients with end-stage ILD.

Nintedanib and pirfenidone attenuate fibroblast activity and inhibit myofibroblast differentiation and potentially angiogenesis. Therefore, the administration of antifibrotic drugs prior to LTx may theoretically lead to an increased risk of bronchial anastomotic complications, bleeding, and impaired wound healing [11,26]. However, experiences with ILD patients undergoing LTx are mostly limited to data in IPF patients, where antifibrotic drugs were used as monotherapies [9,10,11,12,13]. In line with previous studies of patients with IPF undergoing LTx with antifibrotic drugs our data support that antifibrotics as well as combined antifibrotic and immunosuppressant drug therapy is safe in patients with PPF and IPF listed for lung transplantation [9,10,11,12,13]. This is of interest since nintedanib and pirfenidone might reduce acute exacerbations in PPF and attenuate further disease progression [8,27].

Furthermore, beneficial effects of pretreatment with antifibrotics and combination of antifibrotics with immunosuppressive drugs were observed. In both subgroups, the length of mechanical ventilation was shorter. In addition, occurrence of PGD was significantly decreased in patients with lower BMI, reduced intraoperative blood loss and pretreatment with antifibrotic monotherapy as well as combination of antifibrotic drugs and immunosuppressive therapy according to multivariate regression analysis. This is of interest since several risk factors for the development of severe PGD have been described. PGD is presumed to be a consequence of ischemia-reperfusion injury as well as inflammatory events [28,29]. Nintedanib exerts a potent inhibitory activity on the proliferation and migration of lung fibroblasts mainly through the downstream of growth factors, whereas pirfenidone inhibits inflammatory responses mainly through suppression of tumor necrosis factor (TNF)-α, an early mediator of inflammation [30]. In addition, nintedanib has also shown anti-inflammatory effects in various experimental and clinical settings [31,32,33,34]. The described pleiotropic properties of antifibrotics might potentially lead to less deterioration of pulmonary function in the posttransplant period by suppressing acute inflammation in lung ischemia-reperfusion injury, the main cause of PGD.

Interestingly, the patients of our study cohort pretreated with antifibrotics developed significantly less de novo DSA and tended to have reduced occurrence of acute cellular rejections within 30 days after LTX after applying multivariate regression analysis for various variables. Nintedanib and pirfenidone block T-cell activation, which are important regulators of rejection, inhibit the release of cytokines and reduce neutrophil chemotaxis, indicating important graft protective effects [32,33,35,36]. In addition, pirfenidone treatment results in reduced germinal center B cells and T-follicular helper cell frequencies [37]. However, it is hypothesized that due to the short half-lives of antifibrotic agents the potential perioperative harmful effects are reduced, but also attenuate long-term beneficial immunomodulatory effects [9].

So far, no data regarding the effects of antifibrotics as well as combined antifibrotic and immunosuppressant drug therapy in patients with end-stage ILD undergoing LTx are available. Prior studies mainly focused on patients with IPF [10,11,12]. The results of our study should be interpreted with caution and in view of the study design and its limitations, which include a retrospective, single-center setting and a heterogeneous group of end-stage lung diseases with a high portion of patients with PPF. Although severity of disease, comorbidities and LAS score between the groups didn’t differ, a higher proportion of women and people of younger age were seen in the group pretreated with immunosuppressive drugs and suffering from systemic autoimmune disease and pulmonary hypertension. In addition, allograft related factors (e.g., age or compatibility) were not taken into account completely and antifibrotics were used alone or in combination with other ILD specific medication in a small cohort of the study. Therefore, the beneficial effect on transplant outcome cannot solely attributed to antifibrotics and has to be regarded in the context of previous or simultaneous measures and perioperative conditions.

In conclusion, use of antifibrotics and combined antifibrotic and immunosuppressant drug therapy in patients with end-stage ILD until the moment of LTx proved to be safe, having no significant effects on surgical complications, bleeding or wound healing disorders. Furthermore, patients with pretreatment of antifibrotics as well as combined antifibrotic and immunosuppressant drug therapy had reduced length of mechanical ventilation, improved primary graft function and lower prevalence of de novo donor-specific HLA-antibodies. This is a hypothesis-generating observation warranting further studies.

## Figures and Tables

**Table 1:** Baseline characteristics of patients with end-stage ILD undergoing LTX at the time of entering the waiting list.

	All (*n* = 286)	Antifibrotic Monotherapy (*n* = 82)	Antifibrotic Treatment + Immunosuppressive Drugs (*n* = 33)	Immunosuppressive Therapy (*n* = 147)	w/o Specific Treatment (*n* = 24)	*p*-Value
Age (years)	56.8 ± 8.8	59.8 ± 8.1	59.9 ± 6.2	55.2 ± 8.2	52.7 ± 12.9	<0.001
Sex (male), *n* (%)	181 (63.3)	65 (79.3)	21 (63.6)	79 (53.7)	16 (66.7)	0.001
BMI (kg/m^2^)	24.3 ± 3.7	23.6 ± 3.6	23.7 ± 3.5	24.9 ± 3.7	24.5 ± 3.6	0.061
IPF, *n* (%)	116 (40.6)	70 (85.4)	12 (36.4)	26 (17.7)	8 (33.3)	0.001
Smoker Status						
Ex-smoker	157 (54.9)	54 (66.7)	22 (66.7)	65 (44.5)	16 (66.7)	0.003
Pack years	20.9 ± 18.7	22.0 ± 12.5	19.2 ± 22.0	20.3 ± 20.9	21.8 ± 22.4	0.924
Lung function						
FVC, %predicted	44.4 ± 16.4	46.5 ± 16.4	42.0 ± 13.6	43.6 ± 15.4	44.9 ± 24.6	0.492
TLC, %predicted	54.3 ± 15.7	53.9 ± 16.0	54.3 ± 15.8	54.4 ± 15.1	54.7 ± 19.2	0.995
DLCO, %predicted ^a^	21.3 ± 8.1	21.4 ± 7.9	22.0 ± 9.1	20.7 ± 7.2	23.6 ± 12.1	0.717
6MWD (m) ^b^	250.1 ± 124.9	245.9 ± 135.1	240.9 ± 151.3	247.6 ± 112.9	202.9 ± 116.9	0478
pCO_2_ (mmHg)	47.4 ± 12.7	46.6 ± 11.5	47.0 ± 9.6	47.2 ± 13.6	52.1 ± 13.5	0.297
Pulmonary hypertension, *n* (%)	209 (73.6)	61 (75.3)	26 (78.8)	103 (70.5)	19 (79.2)	0.688
Mean PAP ± SD mmHg)	26.8 ± 10.5	26.6 ± 9.3	26.3 ± 9.4	26.7 ± 10.9	29.0 ± 13.3	0.768
GAP-ILD score	5.0 ±1.2	5.2 ± 1.2	5.2 ± 1.1	4.9 ± 1.1	4.9 ± 1.2	0.055
Lung Allocation Score	47.6 ± 15.9	48.2 ± 15.5	45.3 ± 11.8	47.3 ± 16.3	51.0 ± 19.9	0.577

Data are presented as mean ± standard deviation and number and percentage, respectively. Definition of abbreviations: 6MWD, 6-min walk distance; BMI, body mass index, DLCO, diffusion capacity for carbon monoxide; FVC, forced vital capacity; GAP, gender-age-lung physiology; IPF, idiopathic pulmonary fibrosis; pCO_2_, partial pressure of carbon dioxide; PAP; pulmonary arterial pressure (mean ± standard deviation); ILD, interstitial lung disease; LAS, lung allocation score; TLC, total lung capacity. ^a^ Available in 136 patients (49/11/64/12). ^b^ Available in 230 patients (70/26/115/19).

**Table 2:** Perioperative course, lung donor parameters, ICU parameters and surgery-related complications of patients with PPF and IPF undergoing LTx.

	All (*n* = 286)	Antifibrotic Monotherapy (*n* = 82)	Antifibrotic Treatment + Immunosuppressive Drugs (*n* = 33)	Immuno- Suppressive Therapy (*n* = 147)	w/o Specific Treatment (*n* = 24)	*p*-Value
Preoperative						
Mechanical Ventilation, *n* (%)	15 (5.2)	5 (6.1)	0 (0.0)	9 (6.1)	1 (4.2)	0.591
ECMO, veno-venous, *n* (%)	22 (7.7)	4 (4.9)	1 (3.1)	13 (8.9)	4 (16.7)	0.199
Cold ischemia time (hours)						
SLTx	7.7 ± 2.4	8.0 ± 2.6	8.2 ± 3.0	7.2 ± 2.0	8.1 ± 2.5	0.716
BLTx, right side	7.1 ± 2.0	6.6 ± 1.8	6.7 ± 2.0	7.3 ± 2.1	8.2 ± 2.2	0.016
BLTx, left side	7.2 ± 2.3	6.6 ± 2.1	6.3 ± 2.0	7.6 ± 2.3	7.6 ± 2.8	0.003
Donor age (years)	46.6 ± 16.4	46.0 ± 16.2	45.9 ± 15.6	47.7 ± 16.4	42.4 ± 18.8	0.493
Donor BMI (kg/m^2^)	25.2 ± 3.6	24.8 ± 4.1	25.2 ± 3.5	25.4 ± 3.6	25.0 ± 2.4	0.712
Donor TLC (l)	6.5 ± 1.2	6.6 ± 1.1	6.6 ± 1.0	6.4 ± 1.2	6.4 ± 1.3	0.557
PaO_2_ at 100% FiO_2_ (mmHg)	443.6 ± 72.8	445.8 ± 71.3	432.6 ± 96.3	442.3 ± 68.7	459.0 ± 66.9	0.585
Intraoperative						
Operation time (hours) BLTx	5.8 ± 1.2	5.5 ± 0.8	5.5 ± 0.9	5.9 ± 1.3	5.7 ± 1.0	0.315
Operation time (hours) SLTx	2.7 ± 0.6	2.8 ± 0.8	2.3 ± 0.3	2.7 ± 0.6	2.7 ± 0.2	0.554
ECMO, veno-venous, *n* (%)	23 (8.1)	4 (4.9)	1 (3.1)	15 (10.3)	3 (12.5)	0.301
ECMO, veno-arterial, *n* (%)	157 (55.3)	42 (51.2)	18 (56.3)	85 (58.2)	12 (50.0)	0.718
Intra-operative blood loss (ml)	2861.5 ± 2235.0	2105.7 ± 1409.7	2481.8 ± 1432.5	3238.6 ± 2532.2	3687.5 ± 2758.9	<0.001
Surgery-related complications						
Anastomotic complications, *n* (%) *	15 (5.3)	6 (7.3)	0 (0.0)	8 (5.5)	1 (4.2)	0.503
Operative revision, patients, *n* (%) **	68 (23.8)	15 (18.3)	5 (15.2)	40 (27.2)	8 (33.3)	0.185
Due to bleeding, *n* (%)	47 (16.4)	7 (8.5)	5 (15.2)	29 (19.7)	6 (25.0)	0.083
Due to wound dehiscence, hernia, fistula, torsion, *n* (%)	23 (8.0)	8 (9.8)	0 (0.0)	13 (8.8)	2 (8.3)	0.288
Drainage of effusion, *n* (%)	24 (8.4)	5 (6.1)	2 (6.1)	15 (10.2)	2 (8.3)	0.745
Pneumothorax, *n* (%)	16 (5.6)	3 (3.7)	1 (3.0)	12 (8.2)	0 (0.0)	0.343
Postoperative						
ECMO therapy						
Veno-venous, *n* (%)	37 (13.0)	6 (7.3)	2 (6.3)	25 (17.1)	4 (16.7)	0.103
Veno-arterial, *n* (%)	16 (5.6)	3 (3.7)	2 (6.3)	10 (6.8)	1 (4.2)	0.813
Re-intubation rate, *n* (%)	26 (9.1)	7 (8.5)	2 (6.1)	17 (11.6)	0 (0.0)	0.328
Use of inhaled nitric oxid, *n* (%)	153 (53.5)	48 (58.5)	14 (42.4)	77 (52.4)	14 (58.3)	0.436
Mechanical ventilation (hours) ^a^	73.0 (7.0–1128.0)	59.1 (9.2–1128.0)	57.4 (10.0–814.0)	98.0 (10.3–792.0)	69.3 (7.0–808.8)	0.019
PGD T72, grade	1.3 ± 1.0	0.9 ± 0.9	0.8 ± 0.1	1.5 ± 1.1	1.7 ± 1.0	<0.001
T72 Grade 3, *n* (%)	51 (18.4)	6 (7.5)	1 (3.0)	37 (26.4)	7 (29.2)	<0.001
Length of ICU stay (days)	22.1 ± 34.6	18.1 ± 42.0	14.0 ± 14.7	25.6 ± 34.0	25.6 ± 26.4	0.196

Data are presented as mean ± standard deviation and number and percentage, respectively. Definition of abbreviations: BLTx, bilateral lung transplantation; BMI, body mass index; ECMO, extracorporeal membrane oxygenation, ICU, intensive care unit; IPF, idiopathic pulmonary fibrosis; PaO_2_, arterial partial pressure of oxygen; PGD, primary graft dysfunction; PPF, progressive pulmonary fibrosis; SLTx, single lung transplantation; T72, PGD at 72 h after transplantation; TLC, total lung capacity. * Anastomotic complications include anastomosis insufficiency and anastomosis necrosis. ** Operative revision includes hematothorax, wound dehiscence, hernia, fistula and torsion. Three patients suffered from more than one complication. ^a^ presented as median and range.

**Table 3:** Univariate regression (UR, Pearson correlation, coefficient r) and multivariate linear regression (MLR, coefficient beta) analysis factors associated with grade of PGD after 72 h.

All Patients (*n* = 286)	Grade of PGD			
	UR		MLR	
	*p*-Value	Coeff. r	*p*-Value	Coeff. Beta
Gender (m = 1)	0.042	0.123	0.033	0.275
IPF (1) vs. Non-IPF (0)	0.017	−0.143	0.205	0.198
CTD-ILD	0.887	0.009	0.160	−0.285
Age for GAP ILD	0.001	−0.203	0.232	−0.009
SLTX (0) vs. BLTX (1)	0.088	0.103	0.952	−0.006
Use of ECMO before LTX	<0.001	0.222	0.077	0.703
BMI	0.072	0.123	0.022	0.038
LAS Score	0.003	0.178	0.945	<0.001
Time from diagnosis to LTX (months)	0.043	0.122	0.149	0.012
Intraoperative blood loss (ml)	<0.001	0.282	0.003	0.181
Antifibrotics *	<0.001	−0.237	0.011	−0.532
Antifibrotics and immunosuppression before LTX	0.012	−0.151	0.025	−0.519
Steroid (Prednisolone > 5 mg) before LTX	0.036	0.126	0.246	−0.240
Combined Immunosuppression before LTX **	0.024	0.135	0.182	0.255
w/o specific treatment before LTX	0.039	0.124	0.837	−0.049

Definition of abbreviations: BLTx, bilateral lung transplantation; BMI, body mass index; CTD-ILD, connective tissue disease-related interstitial lung disease; ECMO, extracorporeal membrane oxygenation; IPF, idiopathic pulmonary fibrosis; LAS, lung allocation score; LTx, lung transplantation; PAP; pulmonary arterial pressure; PGD, primary graft dysfunction; SLTx, single lung transplantation; T72, PGD at 72 h after transplantation. * Nintedanib or pirfenidone, ** Prednisolone with other immunosuppressant (mycophenolate, methotrexate, azathioprine or others (see Table S1)).

**Table 4:** Immunology and survival of patients with PPF and IPF undergoing LTX.

	All (*n* = 286)	Antifibrotic Monotherapy (*n* = 82)	Antifibrotic Treatment + Immunosuppressive Drugs (*n* = 33)	Immuno- Suppressive Therapy (*n* = 147)	w/o Specific Treatment (*n* = 24)	*p*-Value
Episode of acute cellular rejection (≥A1)						
Within 30 days after LTx, *n* (%) ^a^	34 (13.5)	6 (7.7)	5 (15.6)	17 (13.8)	6 (31.6)	0.057
Within 1 year after LTx, *n* (%)	46 (19.4)	13 (21.0)	5 (21.7)	22 (16.9)	6 (28.6)	0.549
De novo donor specific HLA-antibodies (DSA)						
Within 30 days after LTx, *n* (%) ^b^; (class I/class II/class I + II, *n*)	63 (23.2) (26/25/12)	10 (12.5) (5/4/1)	5 (15.2) (3/1/1)	40 (29.6) (16/15/9)	8 (34.8) (2/5/1)	0.009 0.732
Within 1 year after LTx, *n* (%) ^c^; (class I/class II/class I + II, *n*)	77 (31.0) (31/35/11)	14 (21.5) (5/9/0)	5 (26.3) (3/1/1)	50 (35.2) (21/20/9)	8 (34.8) (2/5/1)	0.249 0.498
Bronchoalveolar Lavage (BAL)						
Neutrophilia within 3 months after LTx ^d^ (%)	8.4 ± 14.9	6.9 ± 8.9	7.2 ± 13.0	10.1 ± 18.3	4.7 ± 7.9	0.523
Neutrophilia > 15%, *n* (%)	18 (15.3)	2 (10.0)	2 (20.0)	13 (17.1)	1 (8.3)	0.801
Neutrophilia within 6 months after LTx (%)	5.1 ± 10.5	4.6 ± 5.8	5.4 ± 12.3	4.8 ± 10.1	7.5 ± 19.3	0.806
30-days mortality, *n* (%)	6 (2.1)	1 (1.2)	0 (0.0)	5 (3.4)	0 (0.0)	0.701
ICU-mortality, *n* (%)	8 (2.8)	2 (2.4)	0 (0.0)	6 (4.1)	0 (0.0)	0.749
1-year mortality, *n* (%)	24 (4.2)	6 (7.3)	1 (3.0)	16 (10.9)	1 (4.2)	0.516

Data are presented as number and percentage and mean ± standard deviation, respectively. BAL, bronchoalveolar lavage; DSA, de novo donor-specific antibodies; ICU, intensive care unit; LTx, lung transplantation. ^a^ Available in 252 patients (78/32/123/19). ^b^ Available in 208 patients (80/33/135/23). ^c^ Available in 248 patients (65/19/142/22). ^d^ Available in 149 patients (44/16/77/12).

**Table 5:** COX regression analysis (death 0, survival 1) during the study period with at least one year follow-up.

All Patients *n* = 286				
	*p*-Value	Coeff. ß	Odd Ratio	CI
Gender (m = 1)	0.174	0.431	1.538	0.827–2.861
IPF (1) vs. Non-IPF (0)	0.003	1.148	3.152	1.463–6.790
CTD-ILD	0.774	0.168	1.183	0.375–3.732
Age (years)	0.004	0.070	1.072	1.022–1.124
SLTX (0) vs. BLTX (1)	0.859	0.038	0.859	0.685–1.573
Use of ECMO before LTX	0.081	−1.375	0.253	0.060–1.068
Mean PAP (mmHg)	0.222	−0.018	0.982	0.954–1.011
BMI	0.054	0.081	1.084	0.998–1.177
LAS Score	0.004	0.031	1.032	1.010–1.054
Time from diagnosis to LTX (months)	0.103	0.023	1.023	0.995–1.052
Intraoperative blood loss (ml)	0.006	<0.001	1.000	1.000–1.000
Antifibrotics *	0.178	−0.673	0.510	0.192–1.358
Antifibrotics and immunosuppression before LTX	0.181	−0.974	0.388	0.097–1.556
Steroid (Prednisolone > 5 mg) before LTX	0.597	0.246	1.278	0.514–3.178
Combined Immunosuppression before LTX **	0.448	−0.331	0.718	0.306–1.686
w/o specific treatment before LTX	0.884	0.078	1.081	0.378–3.091

Definition of abbreviations: BLTx, bilateral lung transplantation; BMI, body mass index; CTD-ILD, connective tissue disease-related interstitial lung disease; ECMO, extracorporeal membrane oxygenation; IPF, idiopathic pulmonary fibrosis; LAS, lung allocation score; LTx, lung transplantation; PAP; pulmonary arterial pressure; SLTx, single lung transplantation. * Nintedanib or pirfenidone, ** Prednisolone with other immunosuppressant.

## Data Availability

All relevant data are within the manuscript and its Supporting Information files.

## Supplementary Materials

The following supporting information can be downloaded at: https://www.mdpi.com/article/10.3390/jcm12154996/s1, Figure S1: Kaplan–Meier survival in patients undergoing lung transplantation with different previous treatment regimens; Table S1: End-stage ILD of the study cohort and concomitant immunosuppressive and/or antifibrotic therapy prior to LTx; Table S2: Comorbidities assessed during transplant evaluation.

## Abbreviations

AZA	azathioprine
BLTx	bilateral lung transplantation
BMI	body mass index
CTD-ILD	connective tissue disease-related ILD
DSA	donor specific antibodies
ECMO	extracorporeal membrane oxygenation
EMA	European Medicines Agency
FDA	Food and Drug Administration
GAP	gender-age-physiology
HP	chronic hypersensitivity pneumonitis
ICU	intensive care unit
IPF	idiopathic pulmonary fibrosis
LAS	lung allocation score
LTx	lung transplantation
MMF	mycophenolate mofetil
NSIP	non-specific interstitial pneumonia
PAP	pulmonary arterial pressure
PGD	primary graft dysfunction
PPF	progressive pulmonary fibrosis
SD	standard deviation
SLTx	single lung transplantation
SSc-ILD	systemic sclerosis-associated ILD
TBB	transbronchial biopsies
uILD	unclassifiable pulmonary fibrosis
UIP	usual interstitial pneumonia
6MWD	6-minute walking distance
