# Supplementary material for: Impact of ILD-Specific Therapies on Perioperative Course in Patients with Progressive Interstitial Lung Disease Undergoing Lung Transplantation
Source: J Clin Med. 2023 Jul 29;12(15):4996. doi: 10.3390/jcm12154996 (PMC10419359; doi:10.3390/jcm12154996)

Supplemental Material

Impact of ILD-specific therapies on perioperative course in patients with progressive interstitial lung disease undergoing lung transplantation

Table S1. End-stage ILD of the study cohort and concomitant immunosuppressive and/or antifibrotic therapy prior to LTx.

	**All (n=286)**	**Idiopathic pulmonary fibrosis (n= 116)**	**Hypersensitivity pneumonitis (n=65)**	**unclassified pulmonary fibrosis (n=20)**	**Non-specific interstitial pneumonia (n=37)**	**Connective tissue disease-associated ILD (n=31)**	**Rare ILDs* (n=17)**
Pirfenidone, n (%)	45 (12.9)	38 (32.8)	2 (3.1)	0 (0.0)	2 (5.4)	0 (0.0)	3 (17.6)
Nintedanib, n (%)	37 (15.7)	32 (27.6)	1 (1.5)	2 (10.0)	0 (0.0)	1 (3.2)	1 (5.9)
Combination of antifibrotics with immunosuppressive drugs, n (%)**	33 (11.5)	12 (10.3)	7 (10.8)	5 (25.0)	2 (5.4)	6 (19.4)	1 (5.9)
Corticosteroid monotherapy, n (%)	80 (28.0)	19 (16.4)	22 (33.8)	8 (40.0)	17 (45.9)	10 (32.3)	4 (23.5)
Corticosteroids + MMF, n (%)	26 (9.1)	4 (3.4)	10 (15.4)	0 (0.0)	6 (16.2)	6 (19.4)	0 (0.0)
Corticosteroids + AZA, n (%)	32 (11.2)	3 (2.6)	18 (27.7)	0 (0.0)	7 (18.9)	4 (12.9)	0 (0.0)
Immunosuppressive monotherapy, n (%)***	9 (3.1)	0 (0.0)	2 (3.1)	0 (0.0)	2 (5.4)	3 (9.7)	2 (11.8)
w/o specific treatment	24 (8.4)	8 (6.9)	3 (4.6)	5 (25.0)	1 (2.7)	1 (3.2)	6 (35.3)

Definition of abbreviations: AZA, azathioprine; ILD, interstitial lung disease; MMF, mycophenolate mofetil. * Respiratory bronchiolitis-interstitial lung disease, eosinophilic pneumonia, acute interstitial pneumonia, desquamative interstitial pneumonia ** Combination of antifibrotics with corticosteroid ± mycophenolate mofetil or ± azathioprine. *** MMF or methotrexate or AZA.

Table S2. Comorbidities assessed during transplant evaluation.

	All (n=286)	Antifibrotic monotherapy (n= 82)	antifibrotic treatment + immunosuppressive drugs (n=33)	Immuno-suppressive therapy (n=147)	w/o specific treatment (n=24)	p-value
Arterial hypertension, n (%)	91 (31.8)	29 (35.4)	10 (30.3)	44 (29.9)	8 (33.3)	0.848
Diabetes mellitus, n (%)	28 (9.8)	6 (7.3)	2 (6.1)	15 (10.2)	5 (20.8)	0.251
Hyperlipoproteinemia, n (%)	35 (12.2)	14 (17.1)	4 (12.1)	13 (8.8)	4 (16.7)	0.243
Coronary artery disease, n (%)	58 (20.3)	24 (29.3)	4 (12.1)	28 (19.0)	2 (8.3)	0.063
History of revascularization, n (%)	33 (11.5)	18 (22.0)	1 (3.0)	12 (8.2)	2 (8.3)	**0.007**
Connective tissue disease-related interstitial lung disease, n (%)	31 (10.8)	1 (1.2)	6 (18.2)	23 (15.6)	1 (4.2)	<0.001
Systemic sclerosis, n (%)	13 (41.9)	0 (0.0)	1 (16.7)	11 (47.8)	1 (100.0)	<0.001
Osteoporosis, n (%)	23 (8.0)	6 (7.3)	2 (6.1)	13 (8.8)	2 (8.3)	0.985
Obstructive sleep apnea syndrome, n (%)	21 (7.3)	7 (8.5)	4 (12.1)	10 (6.8)	0 (0.0)	0.370
Gastroesophageal reflux disease, n (%)	35 (12.2)	11 (13.4)	4 812.1)	16 (10.9)	4 (16.7)	0.792
Atrial fibrillation, n (%)	12 (4.2)	2 (2.4)	1 (3.0)	7 (4.8)	2 (8.3)	0.554
Acetylsalicylic acid, n (%)	78 (27.3)	38 (46.3)	6 (18.2)	26 (17.7)	8 (33.3)	<0.001
Vitamin K antagonist, n (%)	25 (8.7)	5 (6.1)	3 (9.1)	16 (10.9)	1 (4.2)	0.629

Data are presented as number and percentage, respectively.

Figure S1. Kaplan–Meier survival in patients undergoing lung transplantation with different previous treatment regimens.

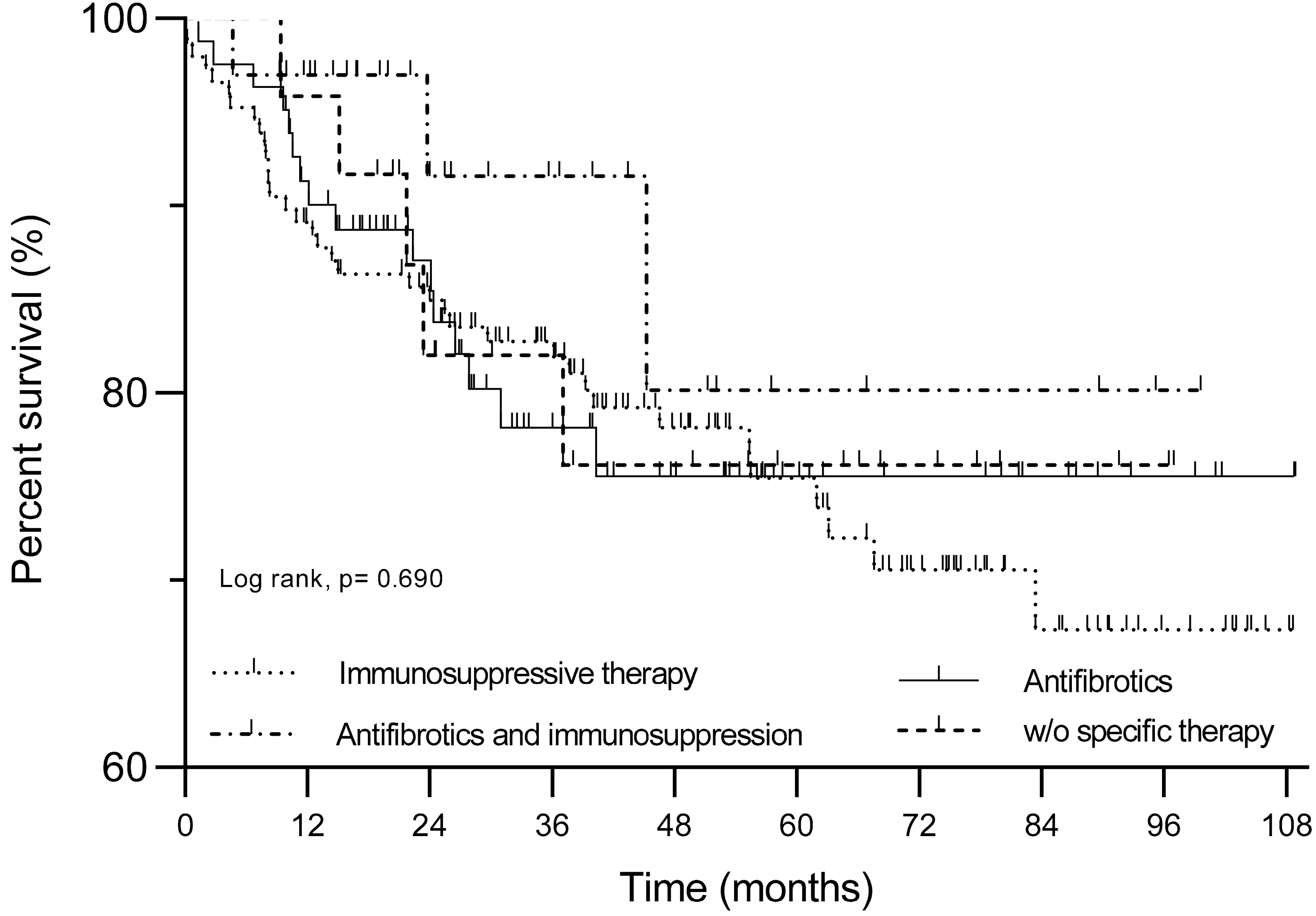

Supplement: Supplementary file 1 [file jcm-12-04996-s001.zip › jcm-2455244-supplementary.pdf]
